# Validity and reliability of the Dutch STarT MSK tool in patients with musculoskeletal pain in primary care physiotherapy

**DOI:** 10.1371/journal.pone.0248616

**Published:** 2021-03-18

**Authors:** Anke G. van den Broek, Corelien J. J. Kloek, Martijn F. Pisters, Cindy Veenhof

**Affiliations:** 1 Department of Rehabilitation, Physical Therapy Science and Sports, Rudolf Magnus Institute of Neurosciences, University Medical Center Utrecht, Utrecht University, Utrecht, The Netherlands; 2 Expertise Center Healthy Urban Living, Research Group Innovation of Human Movement Care, University of Applied Sciences Utrecht, Utrecht, The Netherlands; 3 Center for Physical Therapy Research and Innovation in Primary Care, Julius Health Care Centers, Utrecht, The Netherlands; 4 Research Group Empowering Healthy Behaviour, Fontys University of Applied Sciences, Eindhoven, The Netherlands; University of Pittsburgh, UNITED STATES

## Abstract

**Objective:**

To evaluate the validity and reliability of the Dutch STarT MSK tool in patients with musculoskeletal pain in primary care physiotherapy.

**Methods:**

Physiotherapists included patients with musculoskeletal pain, aged 18 years or older. Patients completed a questionnaire at baseline and follow-up at 5 days and 3 months, respectively. Construct validity was assessed by comparing scores of STarT MSK items with reference questionnaires. Pearson’s correlation coefficients were calculated to test predefined hypotheses. Test-retest reliability was evaluated by calculating quadratic-weighted kappa coefficients for overall STarT MSK tool scores (range 0–12) and prognostic subgroups (low, medium and high risk). Predictive validity was assessed by calculating relative risk ratios for moderate risk and high risk, both compared with low risk, in their ability to predict persisting disability at 3 months.

**Results:**

In total, 142 patients were included in the analysis. At baseline, 74 patients (52.1%) were categorised as low risk, 64 (45.1%) as medium risk and 4 (2.8%) as high risk. For construct validity, nine of the eleven predefined hypotheses were confirmed. For test-retest reliability, kappa coefficients for the overall tool scores and prognostic subgroups were 0.71 and 0.65, respectively. For predictive validity, relative risk ratios for persisting disability were 2.19 (95% CI: 1.10–4.38) for the medium-risk group and 7.30 (95% CI: 4.11–12.98) for the high-risk group.

**Conclusion:**

The Dutch STarT MSK tool showed a sufficient to good validity and reliability in patients with musculoskeletal pain in primary care physiotherapy. The sample size for high-risk patients was small (n = 4), which may limit the generalisability of findings for this group. An external validation study with a larger sample of high-risk patients (≥50) is recommended.

## Introduction

Musculoskeletal conditions, such as low-back pain, neck pain, osteoarthritis and rheumatoid arthritis, are the most common cause of long-term pain and impaired physical function [1], and have large impact on health-related quality of life [2]. In the Netherlands the point prevalence of musculoskeletal pain is found to be 53.9%, with low-back, shoulder, neck and knee being the most frequently affected pain sites [3]. With the high impact of chronic musculoskeletal conditions on health care and work-related costs [4–8], there is a need for effective and cost-effective treatment options to manage musculoskeletal pain.

Musculoskeletal pain is predominantly managed in primary care, for example by the physiotherapist. Although current evidence shows positive effects of exercise therapy and psychosocial interventions on pain and function in patients with musculoskeletal pain [9, 10], it needs to be considered that every patient is unique and some patients respond better to certain treatments than others [11]. In order to improve effectiveness on clinical outcomes and cost-effectiveness in the treatment of patients with musculoskeletal pain, a stratified care approach is promising [12]. Within stratified care, treatments are matched to patients based on key characteristics such as biomedical and psychosocial risk factors for poor prognosis. To identify modifiable risk factors for poor prognosis at an early stage and, subsequently, to stimulate that the appropriate stratified care will be applied to patients, a valid and reliable risk stratification tool is required.

The Keele STarT Back-Screening Tool (SBT) is an example of a valid and reliable risk stratification tool developed to allocate primary care patients with low-back pain into three prognostic subgroups (low, moderate and high risk of persisting back pain disability), and to apply the appropriate matched treatment [13]. The original SBT was developed in the United Kingdom (UK) and has been translated into several languages, including Dutch [14]. A stratified care approach (use of the SBT and matched treatments) has demonstrated superior clinical and cost outcomes compared to usual non-stratified primary care in patients with low-back pain within the UK [15, 16]. While the SBT focuses primarily on back pain, there is evidence that different regional musculoskeletal pain presentations share common underlying mechanisms and prognostic factors [17–20]. These studies indicate that a comparable risk stratification tool could be useful for patients with a broader range of musculoskeletal pain presentations.

Recently, a modified, generic version of the SBT was developed for patients with the five most common musculoskeletal pain presentations (i.e., neck, back, shoulder, knee or multisite pain) within the UK [21, 22]. This so-called Keele STarT MSK tool showed a moderate to good predictive ability of the tool’s baseline score for identifying patients who developed persisting disability because of musculoskeletal pain, and subgroup cut-points were comparable across pain sites [22, 23]. Given the promising predictive performance of the STarT MSK tool we believe that the tool could be useful for Dutch clinical practice as well. The initial STarT MSK study that Dunn et al. conducted, included patients who consulted their general practitioner (GP) with musculoskeletal pain [22]. In the Netherlands, physiotherapists in particular are involved in the treatment of patients with musculoskeletal pain. A first step toward identifying whether the STarT MSK tool could be useful for Dutch primary care physiotherapy, is to translate the tool into Dutch and evaluate its measurement properties. Therefore, the aim of this study is to evaluate the validity and reliability of the Dutch STarT MSK tool in patients with musculoskeletal pain in primary care physiotherapy.

## Methods

### Translation of the STarT MSK tool

Prior to the start of this study, we formally translated the original English version of the STarT MSK tool into Dutch using a forward-backward translation method according to the guidelines of Beaton et al. (S1 Appendix) [24]. The Dutch version of the STarT MSK tool is included in S2 Appendix. The original tool is designed for patients to self-administer and is available on request [25].

### Design

In this clinimetric study a prospective observational design was used to evaluate the measurement properties of the Dutch STarT MSK tool. Patients were asked to complete baseline and follow-up questionnaires (5 days and 3 months), and received usual care from their physiotherapist. This study was not subject to the Medical Research Involving Human Subjects Act (WMO) and received a ‘non-WMO’ declaration from the Medical Research Ethics Committee of the University Medical Centre Utrecht, The Netherlands (registration number 18–082).

### Participants

#### Physiotherapists

A total of 65 physiotherapists within the authors’ network were invited to participate in this study. Only physiotherapists who were working in primary care and were seeing at least 1 to 2 patients with musculoskeletal pain for a first consultation per week were eligible to participate in this study.

#### Patients

The inclusion period for patients was February 2018 to May 2018. Patients were eligible for inclusion when (1) they consulted a participating physiotherapist for musculoskeletal pain (i.e., neck, back, shoulder, knee or multisite pain) during the inclusion period, (2) it was the first consultation for the current episode of musculoskeletal pain, (3) they were aged 18 years or older, (4) they were able to read and write in Dutch and (5) they had an email address. Patients were excluded when (1) during the first consultation red flags were found indicating a possible specific underlying pathology (e.g., fracture, infection, tumor, cauda equina) responsible for the musculoskeletal pain, (2) they consulted a physiotherapist for pre- or post-operative rehabilitation related to the musculoskeletal pain presentation, (3) they were diagnosed with inflammatory arthritis, spondyloarthropathy or polymyalgia rheumatica, (4) they experienced pregnancy-related pain problems or (5) they had a cognitive impairment.

### Study procedure

The participating physiotherapists received information about the study procedure during a one-hour in-company instruction. Physiotherapists informed eligible patients about the study and screened them on in- and exclusion criteria. Patients who were willing to participate received an information letter from the physiotherapist. After patients had given permission to be contacted, the researcher (AB) received their contact information from the physiotherapist using a secured messenger service. Patients were then e-mailed by the researcher (AB) and received a link to the informed consent form as part of the baseline questionnaire. The link to the baseline questionnaire was sent within 24 hours after the first consultation with the physiotherapist. Patients could only start filling-in the questionnaire if informed consent was provided. When necessary, a reminder was sent to the patient within 3 days. Respectively 5 days and 3 months after filling in the baseline questionnaire patients received the link to the follow-up questionnaire.

### Measurements

#### Baseline

**Fig 1:**
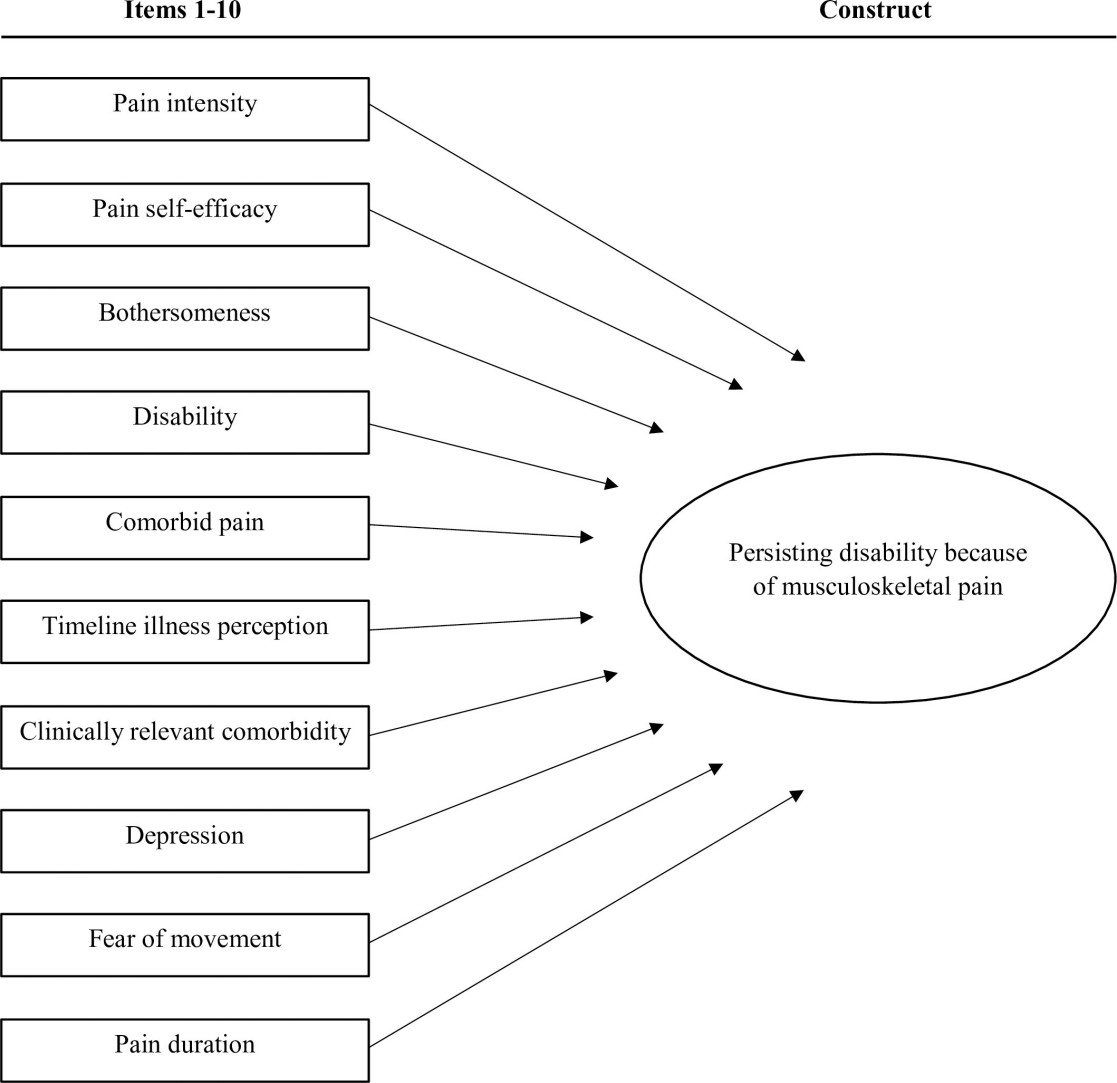
Graphical representation of the STarT MSK tool.

At baseline (T0), general patient characteristics including age, gender, educational level, pain site, pain duration and presence of comorbidity were obtained. The prediction of persisting musculoskeletal-related disability was assessed with the Dutch STarT MSK tool, consisting of 10 independent items that cover biomedical and psychosocial prognostic factors (Fig 1). Subgroup cut-points are 0–4 for low risk, 5–8 for medium risk and 9–12 for high risk, based on an overall score ranging from 0–12 [25]. The average pain in the past week was measured with the 11-point Numeric Pain Rating Scale (NPRS) [26], ranging from 0 (no pain) to 10 (worst possible pain). Pain self-efficacy beliefs were assessed with the Pain Self-Efficacy Questionnaire (PSEQ) [27], consisting of 10 items, each scored on a 7-point Likert scale (0 = not at all confident; 6 = completely confident), with a higher score reflecting stronger self-efficacy beliefs. Disability was measured with the physical functioning subscale of the 36-Item Short Form Health Survey (SF-36 PF) [28], consisting of 10 statements with three answer options varying from ‘Yes, limited a lot’ to ‘No, not limited at all’. Each item is scored 1 to 3 points and the total score was transformed to a 100-point scale, with a higher score indicating better physical functioning. Timeline illness perception was assessed with the timeline question of the Brief Illness Perception Questionnaire (Brief IPQ) [29], ranging from ‘a very short time’ to ‘forever’ on an 11-point scale. Depressive symptoms were measured with the depression subscale of the Hospital Anxiety and Depression Scale (HADS-D) [30], consisting of 7 items, each scored on a 4-point Likert scale, with a higher score reflecting more depressive symptoms. Fear of movement was assessed with the shortened version of the Tampa Scale of Kinesiophobia (TSK-11) [31]. The TSK-11 consists of 11 statements with four answer options varying from ‘strongly disagree’ to ‘strongly agree’. The total score ranges from 11 to 44 points, with a higher score reflecting greater fear of movement.

#### Follow-up

Five days after filling-in the baseline questionnaire patients received a follow-up questionnaire (T1) consisting of the Dutch STarT MSK tool, the NPRS to assess the average pain in the past week and the Global Perceived Effect (GPE) scale [32] to measure recovery. The GPE scale consists of the question ‘To what extend have your complaints improved since filling-in the baseline questionnaire?’, scored on a 7-point Likert scale (1 = completely recovered; 7 = worse than ever).

Three months after filling-in the baseline questionnaire patients received a follow-up questionnaire (T2) consisting of the SF-36 PF to assess disability, the NPRS to assess the average pain in the past week and the GPE scale to measure recovery.

### Main study parameters

The measurement properties construct validity, test-retest reliability and predictive validity were evaluated according to the Consensus-based Standards for the selection of health Measurement Instruments (COSMIN) checklist [33, 34].

#### Construct validity

To evaluate construct validity, we compared scores of the separate STarT MSK items with reference questionnaires. This method was used because the STarT MSK tool is a formative model [35], which means that each item contributes a part of the construct and together they will give a prognosis for persisting musculoskeletal-related disability. As recommended by Mokkink et al. [36], hypotheses were formulated a priori about the expected magnitude of correlations between scores on the instrument (i.e., STarT MSK items) and scores on other instruments. In concordance with Bier et al. [14], reference questionnaires were chosen based on the intended domains being measured using validated Dutch questionnaires. Scores on STarT MSK items 7 and 10 were compared with clinical variables [36], since no validated questionnaires were available. Hypotheses were formulated based on the comparability of the domains being measured. *A priori* we expected a moderate (*r* ≥ 0.3, < 0.5) to high (*r* ≥ 0.5) positive correlation between STarT MSK item 1 with the NPRS, item 6 with the timeline question of the Brief IPQ, item 7 with the single-item question on ‘comorbidity’, item 8 with the HADS-D, item 9 with the TSK-11, item 10 with the single-item question on ‘pain duration’; and a moderate (*r* ≤ -0.3, > -0.5) to high (*r* ≤ -0.5) negative correlation between item 2 with the PSEQ and item 4 with the SF-36 PF [37]. We expected a moderate (*r* ≥ 0.3, < 0.5) to high (*r* ≥ 0.5) positive correlation between the STarT MSK bothersomeness item 3 with the NPRS and a moderate (*r* ≤ -0.3, > -0.5) to high (*r* ≤ -0.5) negative correlation between item 3 with the SF-36 PF, as bothersomeness has been associated with pain and disability [38]. Finally, we expected a low positive correlation (*r* < 0.3) between item 5 and the NPRS, as this item focuses on the location of pain and not on pain intensity.

#### Test-retest reliability

To evaluate test-retest reliability, we assessed the agreement between scores of the Dutch STarT MSK tool on baseline and after 5 days. The time interval was considered long enough to prevent for recall bias, given the large number of questionnaires patients had to complete at baseline. Next, 5 days were considered short enough to prevent substantial improvement [39]. A sensitivity analysis was performed in a subset of patients reporting stable musculoskeletal pain symptoms during the test-retest period. In concordance with the criteria proposed by Bier et al. [14, 40], patients were considered stable between T0 and T1 when they scored ‘slightly worsened’, ‘no change’, or ‘slightly improved’ on the GPE, and had a stable pain score (i.e., the same score on the NPRS plus or minus one point compared with T0).

#### Predictive validity

To evaluate predictive validity, we assessed the ability of the Dutch STarT MSK tool to predict persisting disability at 3 months. Persisting disability was defined as a SF-36 PF score equal to or below the baseline median [13], as standard cutoffs for the SF-36 PF were not available in the literature. We used the SF-36 PF score as poor outcome, because the SF-36 is an assessment tool with good validity regarding its 8 subscales [28, 41]. It was hypothesised that patients with lower baseline scores (i.e., low-risk group compared to medium- or high-risk group and medium-risk group compared to high-risk group) will have better outcomes on the SF-36 PF. As advised by Hill et al. [42], a sensitivity analysis was performed using different subgroup cut-points on the Dutch STarT MSK tool (i.e., low risk 0–3, medium risk 4–7, high risk 8–12; and low risk 0–4, medium risk 5–7, high risk 8–12).

### Sample size

To evaluate construct validity, test-retest reliability and predictive validity, a minimal sample size of 50 patients is advised and a sample size of 100 patients is adequate according to the COSMIN checklist [39, 43]. In this study, we aimed for an adequate sample size of at least 100 patients [43].

### Statistical analysis

Statistical analysis was performed using IBM SPSS Statistics version 25.0 (Armork, New York, USA). Descriptive statistics were calculated for baseline characteristics of the study population, with continuous variables presented using mean and standard deviations. Categorical and nominal/dichotomous data were presented as proportions for each category.

#### Construct validity

Pearson’s correlations were calculated between specific items of the Dutch STarT MSK tool and their reference questionnaires. The construct validity was defined as good if at least 75% of the a priori hypotheses could be confirmed [39].

#### Test-retest reliability

The quadratic-weighted kappa was calculated for overall scores of the Dutch STarT MSK tool and prognostic subgroups, with κ ≤ 0 indicating poor agreement, κ = 0.01–0.20 slight agreement, κ **=** 0.21–0.40 fair agreement, κ **=** 0.41–0.60 moderate agreement, κ **=** 0.61–0.80 substantial agreement and κ = 0.81–1.00 almost perfect agreement [44].

#### Predictive validity

To examine calibration of the STarT MSK tool, we calculated the proportion of patients with persisting disability at 3 months in each risk group. Relative risk ratios (RRs) were calculated for medium risk and high risk, each compared with low risk, in their ability to predict persisting disability at 3 months.

## Results

**Fig 2:**
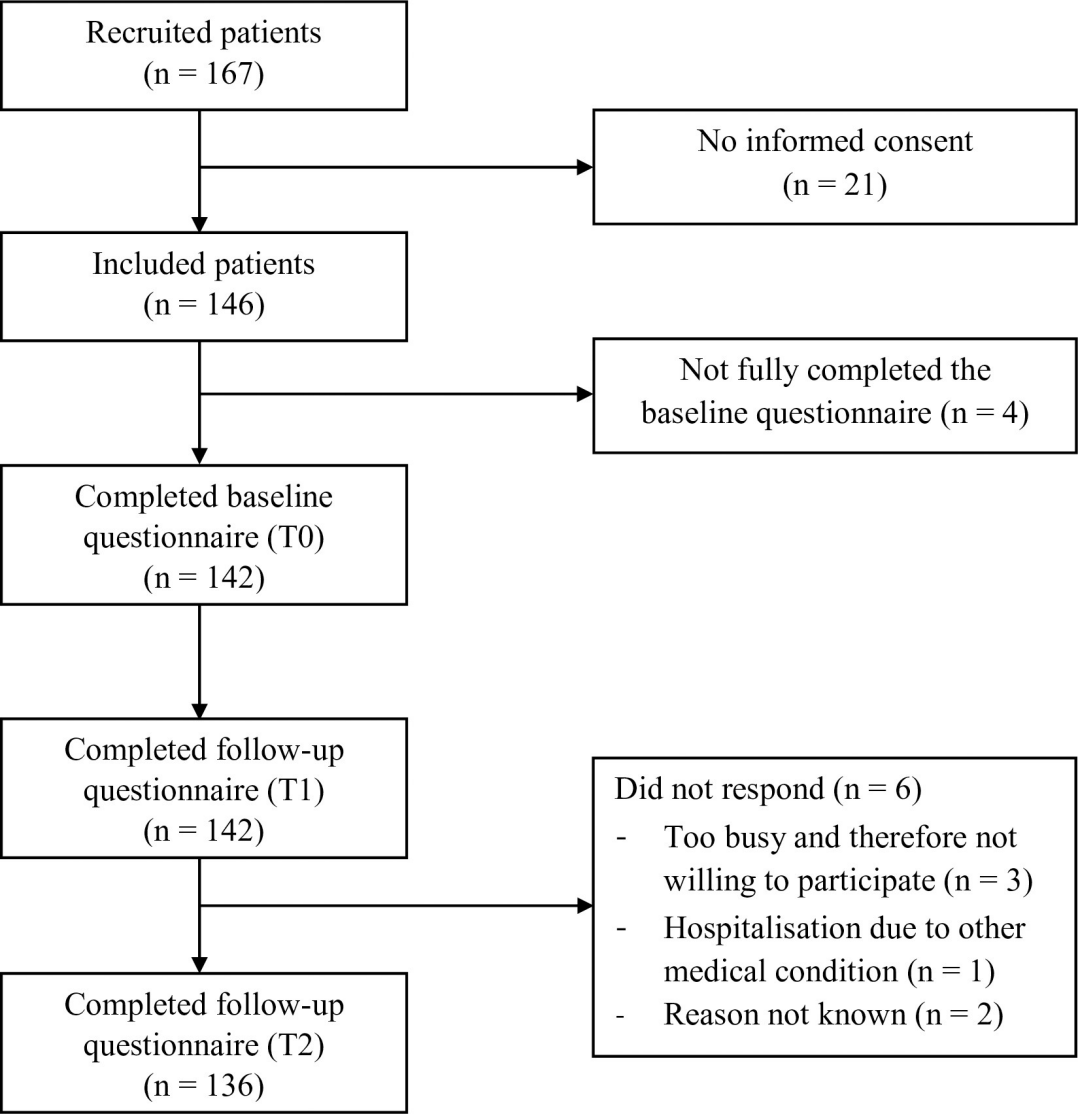
Flowchart for inclusion of patients.

In total, 44 physiotherapists were willing to participate and instructed. A number of 22 physiotherapists from 11 primary care physiotherapy clinics actually recruited patients. Of the participating physiotherapists, the majority was specialised as manual therapist or sports physiotherapist. A total of 167 patients were recruited, of whom 146 patients were included (Fig 2). Four patients were excluded from the analysis, because they did not fully complete the baseline questionnaire. For the follow-up questionnaires at T1 and T2, a 100% and 96% follow-up rate were achieved, respectively.

On average, the time between the first consultation of physiotherapy and completing the baseline questionnaire was 2 days. Baseline characteristics of the study population are presented in Table 1. The mean age of study participants was 48.2 ± 15.6 years (range 18–81), and 57.0% were female. The study population consisted of 142 patients with musculoskeletal pain, of whom 44 patients (31.0%) had back pain, 35 (24.7%) shoulder pain, 28 (19.7%) multisite pain, 25 (17.6%) knee pain and 10 (7.0%) neck pain. Of the patients with multisite pain, the majority (57.1%) reported neck and shoulder pain. At baseline, 74 patients (52.1%) were categorised as low risk, 64 (45.1%) as medium risk and 4 (2.8%) as high risk. Age and gender were comparable across risk groups. For each increase in risk profile, there was a corresponding increase in pain intensity, disability, timeline illness perception, depression and fear of movement.

**Table 1:** Baseline characteristics of the study population*.

	Total	Low risk	Medium risk	High risk
	(n = 142)	(n = 74)	(n = 64)	(n = 4)
Female	81 (57.0)	40 (54.1)	39 (60.9)	2 (50.0)
Age in years	48.2 ± 15.6	47.9 ± 16.8	47.9 ± 14.5	56.8 ± 8.2
Pain site				
Neck	10 (7.0)	6 (8.1)	3 (4.7)	1 (25.0)
Back	44 (31.0)	22 (29.7)	21 (32.8)	1 (25.0)
Shoulder	35 (24.7)	23 (31.1)	12 (18.8)	0 (0.0)
Knee	25 (17.6)	12 (16.2)	12 (18.8)	1 (25.0)
Multisite	28 (19.7)	11 (14.9)	16 (25.0)	1 (25.0)
Episode duration				
<3 months	72 (50.7)	46 (62.2)	25 (39.1)	1 (25.0)
3 to 6 months	26 (18.3)	16 (21.6)	9 (14.1)	1 (25.0)
6 months or longer	44 (31.0)	12 (16.2)	30 (46.9)	2 (50.0)
Presence of comorbidity	59 (41.5)	28 (37.8)	29 (45.3)	2 (50.0)
Dutch STarT MSK tool score^¶^	4.2 ± 2.3	2.4 ± 1.2	6.0 ± 1.0	9.5 ± 0.6
Dutch STarT MSK tool risk profile				
Low	74 (52.1)			
Medium	64 (45.1)			
High	4 (2.8)			
Pain intensity^¶^	5.4 ± 2.0	4.5 ± 2.0	6.2 ± 1.6	8.0 ± 0.0
Mild (0–5)	62 (43.7)	47 (63.5)	15 (23.4)	0 (0.0)
Moderate (6–7)	60 (42.3)	21 (28.4)	39 (60.9)	0 (0.0)
Severe (8–10)	20 (14.1)	6 (8.1)	10 (15.6)	4 (100.0)
Pain self-efficacy^¶^	49.4 ± 9.6	53.5 ± 7.3	45.6 ± 9.4	34.0 ± 9.9
Disability^¶^	75.1 ± 19.5	83.0 ± 13.7	67.2 ± 21.4	56.3 ± 19.7
Timeline illness perception^¶^	4.2 ± 2.9	3.1 ± 2.2	5.3 ± 3.0	7.5 ± 2.1
Depression^¶^	2.3 ± 2.5	1.3 ± 1.8	3.1 ± 2.4	7.3 ± 4.0
Fear of movement^¶^	20.0 ± 5.6	18.5 ± 5.1	21.2 ± 5.4	28.3 ± 7.2

* Values are numbers (percentage) or mean ± standard deviation.

¶ Dutch STarT MSK tool score (0–12); Pain intensity (0–10); Pain self-efficacy (0–60); Disability (0–100); Timeline illness perception (0–10); Depression (0–21); Fear of movement (11–44).

### Construct validity

The highest correlations were found between item 1 with the NPRS and item 10 with the single-item question on ‘pain duration’ (Table 2). The correlations between item 2 with the PSEQ and item 9 with the TSK-11 were lower than hypothesised. Of the predefined hypotheses, 81.8% were confirmed which indicates a good construct validity.

**Table 2:** Pearson’s correlation between the Dutch STarT MSK tool and reference questionnaires.

STarT MSK	Reference questionnaire	A priori	r	Correlation	Expected
Item 1	NPRS	r ≥ 0.3	0.82	High	Yes
Item 2	PSEQ	r ≤ -0.3	-0.22	Low	No
Item 3	NPRS	r ≥ 0.3	0.48	Moderate	Yes
Item 3	SF-36 PF	r ≤ -0.3	-0.42	Moderate	Yes
Item 4	SF-36 PF	r ≤ -0.3	-0.47	Moderate	Yes
Item 5	NPRS	r < 0.3	0.13	Low	Yes
Item 6	Timeline Brief IPQ	r ≥ 0.3	0.57	High	Yes
Item 7	Single item ‘comorbidity’	r ≥ 0.3	0.34	Moderate	Yes
Item 8	HADS-D	r ≥ 0.3	0.39	Moderate	Yes
Item 9	TSK-11	r ≥ 0.3	0.29	Low	No
Item 10	Single item ‘pain duration’	r ≥ 0.3	0.80	High	Yes

r = Pearson’s correlation.

NPRS = Numeric Pain Rating Scale, PSEQ = Pain Self-Efficacy Questionnaire. SF-36 PF = Physical Functioning subscale of the 36-item Short Form Health Survey, IPQ = Illness Perception Questionnaire, HADS-D = depression subscale of the Hospital Anxiety and Depression Scale, TSK-11 = 11-item Tampa Scale of Kinesiophobia.

### Test-retest reliability

In total, 142 patients (100%) completed the Dutch STarT MSK tool at T0 and T1, of whom 77 patients were regarded as stable. On average, the time between T0 and T1 was 7 days. The quadratic-weighted kappa coefficients for the overall STarT MSK tool scores (range 0–12) and prognostic subgroups (low, medium and high risk) were 0.71 and 0.65, respectively, indicating substantial agreement (Table 3). Distribution of prognostic subgroups was skewed due to the very low prevalence of patients at high risk. Test-retest reliability increased to 0.75 for the overall tool scores and decreased to 0.60 for prognostic subgroups, when agreement was calculated in the subset of 77 patients reporting stable musculoskeletal pain symptoms. Of the clinically stable patients, 23.4% were categorised differently within approximately 7 days.

**Table 3:** Quadratic-weighted kappa for the Dutch STarT MSK tool*.

	Test-retest sample (n = 142)	Subset of patients reporting stable symptoms (n = 77)
Overall tool scores (range 0–12)	0.71 (0.62–0.79)	0.75 (0.66–0.83)
Prognostic subgroups (low, medium and high risk)	0.65 (0.54–0.76)	0.60 (0.45–0.76)

* Values are represented as quadratic-weighted kappa coefficients with 95% confidence intervals.

κ ≤ 0: poor agreement, κ = 0.01–0.20: slight agreement, κ = 0.21–0.40: fair agreement, κ = 0.41–0.60: moderate agreement, κ = 0.61–0.80: substantial agreement, κ = 0.81–1.00: almost perfect agreement.

### Predictive validity

In total, 136 patients (96%) completed the follow-up questionnaire at T2, of whom 73 patients were classified as low risk at baseline, 60 as medium risk, and 3 as high risk for persisting disability because of musculoskeletal pain. The mean (SD) SF-36 PF score at follow-up was 88.2 (14.4). The proportion of patients with persisting disability (SF-36 PF ≤ 80.0) was 13.7% in the low-risk group, 30.0% in the medium-risk group, and 100.0% in the high-risk group (Table 4). The RRs for persisting disability were 2.19 (95% CI: 1.10–4.38) for the medium-risk group and 7.30 (95% CI: 4.11–12.98) for the high-risk group, both compared to the low-risk group. The sensitivity analysis using different subgroup cut-points did not resulted in a substantial larger sample of high-risk patients and revealed less sufficient predictive performance of the Dutch STarT MSK tool (S1 Table).

**Table 4:** Proportions and relative risks of persistent disability at 3 months*.

	Proportion (95% CI)^¶^	Relative risk (95% CI)^¶^
Low risk^‡^	13.7 (7.6–23.4)	NA
Medium risk^‡^	30.0 (19.9–42.5)	2.19 (1.10–4.38)
High risk^‡^	100.0 (43.9–100.0)	7.30 (4.11–12.98)

* Persisting disability is defined as a Physical Functioning subscale (SF-36 PF) score equal to or below the baseline median at 3 months.

‡ Low risk (n = 73); Medium risk (n = 60); High risk (n = 3).

¶ Relative risks for medium risk and high risk, each compared with low risk.

CI = confidence interval; NA = not applicable.

## Discussion

The aim of this study was to evaluate the validity and reliability of the Dutch STarT MSK tool in patients with musculoskeletal pain in primary care physiotherapy. The results showed a good construct validity, as 81.8% of the predefined hypotheses were confirmed. Test-retest reliability was substantial for the overall tool scores and prognostic subgroups in the test-retest sample, with quadratic-weighted kappa coefficients of 0.71 and 0.65, respectively. Test-retest reliability remained substantial for the overall scores and decreased slightly to a kappa of 0.60 for prognostic subgroups in the subset of patients reporting stable symptoms between T0 and T1. The predictive validity was sufficient, with RRs for persisting disability of 2.19 (95% CI: 1.10–4.38) for the medium-risk group and 7.30 (95% CI: 4.11–12.98) for the high-risk group, both compared to the low-risk group. The sample size for high-risk patients was small, therefore results for this group should be interpreted with caution.

The risk group distribution in our study was different from the distribution in the UK validation study [22] and STarT MSK pilot cluster Randomised Controlled Trial [23], in which our cohort had a shift toward low risk at the expense of high risk. In the present study, only 4 patients (2.8%) were categorised as high risk, which is significantly lower compared to the UK cohorts (respectively 19.0% and 13.3%). Although the low proportion of high-risk patients might suggest that the Dutch STarT MSK tool is not adequately able to distinguish between medium- and high-risk subgroups, several factors might explain the skewed distribution found in our study. First, we included patients who consulted their physiotherapist for musculoskeletal pain, while in the UK study patients consulted their GP. Most of the participating physiotherapists were specialised as manual therapist or sports physiotherapist. Consequently, it is possible that the more severe psychosomatic cases and potentially high-risk patients were missed, since they possibly visit GPs or specialised psychosomatic physiotherapists. Second, different cultures and health care systems might have contributed to the discrepancy in high-risk patients found between cohorts. Finally, we considered that the original STarT MSK subgroup cut-points might not be appropriate for our clinical population; however the sensitivity analysis with different subgroup cut-points did not result in a substantial larger sample of high-risk patients and revealed less sufficient predictive performance of the tool.

Although the test-retest reliability found in our study suggests that the Dutch STarT MSK tool is able to classify patients into the same prognostic subgroup over time, further research may need to focus on the timing of stratification. We found a quadratic weighted kappa coefficient of 0.65 for prognostic subgroups, indicating substantial agreement. The kappa coefficient decreased to 0.60, indicating moderate agreement, when reliability was calculated using clinically stable patients. It seems more appropriate to consider the test-reliability of prognostic subgroups as substantial since distribution of prognostic subgroups was skewed due to the very low prevalence of patients at high risk, which increases chance agreement and reduces the value of kappa accordingly [45]. Additional analyses showed that 23.4% of the clinically stable patients were categorised differently within approximately 7 days. This finding is in line with a previous finding that changes in SBT categorisation might occur in the first few days after start of initial treatment [46]. Newell et al. found that one third of patients switched SBT risk groups within the 2 days between the initial stratification and after the first treatment. Especially in acute high-risk patients, psychosocial risk factors might be addressed during the first consultation [47], which could influence the results [14]. For example, specific concerns such as the likeliness of a serious underlying pathology and unhelpful beliefs and behaviors such as fear of movement can be addressed during the primary consultation. Despite the test-retest reliability was at an acceptable level, it has to be acknowledged that changes in risk categorisation after the first consultation of physiotherapy could potentially interfere the test-retest reliability. Future research should critically evaluate the most optimal timing of stratification (i.e., at initial assessment or within a few days after first treatment) for successfully predicting persisting musculoskeletal-related disability.

In our study patients received usual care from their physiotherapist. This methodology was chosen, because it most closely resembles how the tool will be used in physiotherapy practice and it seems not ethical to refrain patients from physiotherapy treatment during a 3-month period. Physiotherapy treatment might have influenced the predictive validity of the Dutch STarT MSK tool. In a study regarding changes in SBT categorisation during routine physiotherapy care, 81.8% of the high-risk, 76.0% of the medium-risk and 11.3% of the low-risk patients were categorised differently within a few weeks [48]. In a Danish SBT validation study, difference in risk prediction was observed between the Danish and original UK cohort, in which exposure to physiotherapy treatment was considered to be a confounder [49]. Morso et al. found that patients in the physiotherapy treatment group had a substantially lower risk of persisting disability than the GP group. Therefore, in our study, it is possible that the predictive validity of the Dutch STarT MSK tool might be reduced because of the natural history of the musculoskeletal pain condition being modified by physiotherapy treatment [49]. Despite the possible influence of physiotherapy treatment, the predictive validity showed statistically significant results. However, the confidence intervals of medium- and high risk were wide and show minor overlap, which may indicate a lack of power. The small sample size of high-risk patients and 100% of these patients having persisting disability at 3 months, may have led to an overestimation of the RR for the high-risk group.

To our knowledge, the STarT MSK tool is the only screening tool originally developed to allocate primary care patients with musculoskeletal pain into prognostic subgroups and subsequently stratify for the appropriate matched treatment. The prediction of persisting musculoskeletal-related disability is based on the presence of biomedical and psychosocial prognostic factors at initial assessment. Beneciuk et al. concluded that reliance only on initial (SBT) risk categorisation may have limitations due to changes that might occur following routine physical therapy, and suggested that repeated assessment of risk categorisation can potentially improve prognosis for long-term low-back pain related disability [48]. Given the rapid development of “big data”, this raises the question whether other screening tools, incorporating big data analysis such as machine learning algorithms, may be more adequate to use in targeting treatment to individual patients. The application of machine learning in musculoskeletal physiotherapy is upcoming [50]. However, despite the possible advantages of big data and machine learning algorithms, there are still several challenges that needs to be addressed before further application in health care [51–53]. For now, the Dutch STarT MSK tool seems an easy to use screening tool to start with in physiotherapy practice.

The strength of this study is that we evaluated the measurement properties of the Dutch STarT MSK tool in primary care physiotherapy. In the Netherlands, physiotherapists in particular are involved in the treatment of patients with musculoskeletal pain and therefore are likely the primary users of the tool. Another strength is that we achieved an appropriate sample size (>100 patients) and respectively 100% and 96% follow-up rate. The present study has some limitations. First, the setting in which patients were included might have resulted in the low proportion of high-risk patients and limited generalisability of findings. Second, for feasibility and ethical reasons, patients received usual care from their physiotherapists. Effective physiotherapy treatment could have influenced the predictive validity of the Dutch STarT MSK tool. Third, the sample size for high-risk patients was too small to calculate performance characteristics (i.e., sensitivity and specificity) in order to examine discrimination of the Dutch STarT MSK tool [54]. Finally, we followed the method of Bier et al. [14] and used the guidelines of Cohen [37] as cut-off points for defining the strength of the expected correlations between the STarT MSK items and reference questionnaires. It has to be acknowledged that these cut-off points are arbitrary and there are no widely accepted criteria for defining the strength of a relationship [55]. For the interpretation of complex abstract phenomena, lower correlations are often used as evidence of relationships [56] and therefore the cut-off point of 0.3 seems appropriate for the psychosocial variables. However, for some biomedical variables, a higher cut-off point might have been more appropriate.

This validation study is a first step toward identifying whether the Dutch STarT MSK tool could be useful for physiotherapy practice. According to the psychometric analysis, the tool showed a sufficient to good validity and reliability in patients with musculoskeletal pain in primary care physiotherapy. Based on these results, the tool can be implemented in Dutch physiotherapy practice, in which it can be used to predict persisting disability at an early stage. Because of the small sample size for high-risk patients (n = 4), generalisability of findings may be limited. An external validation study with a larger sample of high-risk patients (≥50) is recommended. Furthermore, the Dutch STarT MSK tool is not just a prognostic screening tool, but can be used to stratify patients for the appropriate matched treatment as well. Matched treatment packages are available for patients with low-back pain [47]. In addition, recently, primary care matching treatment options for patients with the five most common musculoskeletal pain presentations were proposed in a consensus groups study [57]. Future research should focus on whether these proposed matching treatment options are applicable within the Netherlands and particularly the primary care physiotherapy setting. Subsequently, further research is needed to determine whether the entire stratified care approach (use of the STarT MSK tool and matched/targeted treatments) can be effectively used in primary care physiotherapy in the Netherlands.

## Supporting information

S1 Appendix Forward-backward translation method Dutch STarT MSK tool. (PDF)

S2 Appendix The Dutch version of the STarT MSK tool (including scoring method). (PDF)

S1 Table Sensitivity analysis predictive validity using different subgroup cut-points. (PDF)

## References

[1] Woolf AD, Pfleger B. Burden of major musculoskeletal conditions. Bulletin of the World Health Organization. 2003;81(9):646–56. Epub 2004/01/09. 14710506 PMC2572542

[2] Picavet HS, Hoeymans N. Health related quality of life in multiple musculoskeletal diseases: SF-36 and EQ-5D in the DMC3 study. Annals of the rheumatic diseases. 2004;63(6):723–9. Epub 2004/05/14. 10.1136/ard.2003.010769 15140781 PMC1755044

[3] Picavet HS, Schouten JS. Musculoskeletal pain in the Netherlands: prevalences, consequences and risk groups, the DMC(3)-study. Pain. 2003;102(1–2):167–78. Epub 2003/03/07. 10.1016/s0304-3959(02)00372-x . 12620608

[4] Meerding WJ, Bonneux L, Polder JJ, Koopmanschap MA, van der Maas PJ. Demographic and epidemiological determinants of healthcare costs in Netherlands: cost of illness study. BMJ (Clinical research ed). 1998;317(7151):111–5. Epub 1998/07/10. 10.1136/bmj.317.7151.111 9657785 PMC28601

[5] March L, Smith EU, Hoy DG, Cross MJ, Sanchez-Riera L, Blyth F, et al. Burden of disability due to musculoskeletal (MSK) disorders. Best practice & research Clinical rheumatology. 2014;28(3):353–66. Epub 2014/12/08. 10.1016/j.berh.2014.08.002 . 25481420

[6] van der Zee-Neuen A, Putrik P, Ramiro S, Keszei A, de Bie R, Chorus A, et al. Impact of Chronic Diseases and Multimorbidity on Health and Health Care Costs: The Additional Role of Musculoskeletal Disorders. Arthritis care & research. 2016;68(12):1823–31. Epub 2016/04/26. 10.1002/acr.22913 . 27111195

[7] de Vroome EM, Uegaki K, van der Ploeg CP, Treutlein DB, Steenbeek R, de Weerd M, et al. Burden of Sickness Absence Due to Chronic Disease in the Dutch Workforce from 2007 to 2011. Journal of occupational rehabilitation. 2015;25(4):675–84. Epub 2015/03/26. 10.1007/s10926-015-9575-4 . 25804926

[8] de Vries HJ, Reneman MF, Groothoff JW, Geertzen JH, Brouwer S. Self-reported work ability and work performance in workers with chronic nonspecific musculoskeletal pain. Journal of occupational rehabilitation. 2013;23(1):1–10. Epub 2012/06/05. 10.1007/s10926-012-9373-1 22661341 PMC3563949

[9] Babatunde OO, Jordan JL, Van der Windt DA, Hill JC, Foster NE, Protheroe J. Effective treatment options for musculoskeletal pain in primary care: A systematic overview of current evidence. PloS one. 2017;12(6):e0178621. Epub 2017/06/24. 10.1371/journal.pone.0178621 28640822 PMC5480856

[10] Lin I, Wiles L, Waller R, Goucke R, Nagree Y, Gibberd M, et al. What does best practice care for musculoskeletal pain look like? Eleven consistent recommendations from high-quality clinical practice guidelines: systematic review. British journal of sports medicine. 2020;54(2):79–86. Epub 2019/03/04. 10.1136/bjsports-2018-099878 . 30826805

[11] Jellema P, van der Horst HE, Vlaeyen JW, Stalman WA, Bouter LM, van der Windt DA. Predictors of outcome in patients with (sub)acute low back pain differ across treatment groups. Spine. 2006;31(15):1699–705. Epub 2006/07/04. 10.1097/01.brs.0000224179.04964.aa . 16816766

[12] Foster NE, Hill JC, O’Sullivan P, Hancock M. Stratified models of care. Best practice & research Clinical rheumatology. 2013;27(5):649–61. Epub 2013/12/10. 10.1016/j.berh.2013.10.005 . 24315146

[13] Hill JC, Dunn KM, Lewis M, Mullis R, Main CJ, Foster NE, et al. A primary care back pain screening tool: identifying patient subgroups for initial treatment. Arthritis and rheumatism. 2008;59(5):632–41. Epub 2008/04/29. 10.1002/art.23563 . 18438893

[14] Bier JD, Ostelo RWJG, van Hooff ML, Koes BW, Verhagen AP. Validity and Reproducibility of the STarT Back Tool (Dutch Version) in Patients With Low Back Pain in Primary Care Settings. Physical therapy. 2017;97(5):561–70. Epub 2017/03/25. 10.1093/ptj/pzx023 . 28340202

[15] Hill JC, Whitehurst DG, Lewis M, Bryan S, Dunn KM, Foster NE, et al. Comparison of stratified primary care management for low back pain with current best practice (STarT Back): a randomised controlled trial. Lancet (London, England). 2011;378(9802):1560–71. Epub 2011/10/04. 10.1016/S0140-6736(11)60937-9 21963002 PMC3208163

[16] Foster NE, Mullis R, Hill JC, Lewis M, Whitehurst DG, Doyle C, et al. Effect of stratified care for low back pain in family practice (IMPaCT Back): a prospective population-based sequential comparison. Annals of family medicine. 2014;12(2):102–11. Epub 2014/03/13. 10.1370/afm.1625 24615305 PMC3948756

[17] Mallen CD, Peat G, Thomas E, Dunn KM, Croft PR. Prognostic factors for musculoskeletal pain in primary care: a systematic review. The British journal of general practice: the journal of the Royal College of General Practitioners. 2007;57(541):655–61. Epub 2007/08/11. 17688762 PMC2099673

[18] Henschke N, Ostelo RW, Terwee CB, van der Windt DA. Identifying generic predictors of outcome in patients presenting to primary care with nonspinal musculoskeletal pain. Arthritis care & research. 2012;64(8):1217–24. Epub 2012/03/17. 10.1002/acr.21665 . 22422737

[19] Mallen CD, Thomas E, Belcher J, Rathod T, Croft P, Peat G. Point-of-care prognosis for common musculoskeletal pain in older adults. JAMA internal medicine. 2013;173(12):1119–25. Epub 2013/05/24. 10.1001/jamainternmed.2013.962 . 23699833

[20] Croft P, Lewis M, Hannaford P. Is all chronic pain the same? A 25-year follow-up study. Pain. 2003;105(1–2):309–17. Epub 2003/09/23. 10.1016/s0304-3959(03)00246-x . 14499449

[21] Campbell P, Hill JC, Protheroe J, Afolabi EK, Lewis M, Beardmore R, et al. Keele Aches and Pains Study protocol: validity, acceptability, and feasibility of the Keele STarT MSK tool for subgrouping musculoskeletal patients in primary care. Journal of pain research. 2016;9:807–18. Epub 2016/10/30. 10.2147/JPR.S116614 27789972 PMC5072582

[22] Dunn KM, Campbell P, Afolabi EK, Lewis M, van der Windt D, Hill JC, et al. 176. Refinement and validation of the Keele STarT MSK tool for musculoskeletal pain in primary care. Rheumatology. 2017;56(suppl_2):kex062.177–kex062.177. 10.1093/rheumatology/kex062.177

[23] Hill JC, Garvin S, Chen Y, Cooper V, Wathall S, Saunders B, et al. Stratified primary care versus non-stratified care for musculoskeletal pain: findings from the STarT MSK feasibility and pilot cluster randomized controlled trial. BMC family practice. 2020;21(1):30. Epub 2020/02/13. 10.1186/s12875-019-1074-9 32046647 PMC7014664

[24] Beaton DE, Bombardier C, Guillemin F, Ferraz MB. Guidelines for the process of cross-cultural adaptation of self-report measures. Spine. 2000;25(24):3186–91. Epub 2000/12/22. 10.1097/00007632-200012150-00014 . 11124735

[25] Keele University. The Keele STarT MSK tool©2017. Available from: https://www.keele.ac.uk/startmsk/.

[26] Hjermstad MJ, Fayers PM, Haugen DF, Caraceni A, Hanks GW, Loge JH, et al. Studies comparing Numerical Rating Scales, Verbal Rating Scales, and Visual Analogue Scales for assessment of pain intensity in adults: a systematic literature review. Journal of pain and symptom management. 2011;41(6):1073–93. Epub 2011/05/31. 10.1016/j.jpainsymman.2010.08.016 . 21621130

[27] van der Maas LC, de Vet HC, Köke A, Bosscher RJ, Peters ML. Psychometric Properties of the Pain Self-Efficacy Questionnaire (PSEQ). European Journal of Psychological Assessment. 2012.

[28] Aaronson NK, Muller M, Cohen PD, Essink-Bot ML, Fekkes M, Sanderman R, et al. Translation, validation, and norming of the Dutch language version of the SF-36 Health Survey in community and chronic disease populations. Journal of clinical epidemiology. 1998;51(11):1055–68. Epub 1998/11/17. 10.1016/s0895-4356(98)00097-3 . 9817123

[29] de Raaij EJ, Schroder C, Maissan FJ, Pool JJ, Wittink H. Cross-cultural adaptation and measurement properties of the Brief Illness Perception Questionnaire-Dutch Language Version. Manual therapy. 2012;17(4):330–5. Epub 2012/04/10. 10.1016/j.math.2012.03.001 . 22483222

[30] Spinhoven P, Ormel J, Sloekers PP, Kempen GI, Speckens AE, Van Hemert AM. A validation study of the Hospital Anxiety and Depression Scale (HADS) in different groups of Dutch subjects. Psychological medicine. 1997;27(2):363–70. Epub 1997/03/01. 10.1017/s0033291796004382 . 9089829

[31] Woby SR, Roach NK, Urmston M, Watson PJ. Psychometric properties of the TSK-11: a shortened version of the Tampa Scale for Kinesiophobia. Pain. 2005;117(1–2):137–44. Epub 2005/08/02. 10.1016/j.pain.2005.05.029 . 16055269

[32] Kamper SJ, Ostelo RW, Knol DL, Maher CG, de Vet HC, Hancock MJ. Global Perceived Effect scales provided reliable assessments of health transition in people with musculoskeletal disorders, but ratings are strongly influenced by current status. Journal of clinical epidemiology. 2010;63(7):760–6.e1. Epub 2010/01/09. 10.1016/j.jclinepi.2009.09.009 . 20056385

[33] Mokkink LB, Terwee CB, Patrick DL, Alonso J, Stratford PW, Knol DL, et al. The COSMIN study reached international consensus on taxonomy, terminology, and definitions of measurement properties for health-related patient-reported outcomes. Journal of clinical epidemiology. 2010;63(7):737–45. Epub 2010/05/25. 10.1016/j.jclinepi.2010.02.006 . 20494804

[34] Mokkink LB, Terwee CB, Patrick DL, Alonso J, Stratford PW, Knol DL, et al. The COSMIN checklist for assessing the methodological quality of studies on measurement properties of health status measurement instruments: an international Delphi study. Quality of life research: an international journal of quality of life aspects of treatment, care and rehabilitation. 2010;19(4):539–49. Epub 2010/02/20. 10.1007/s11136-010-9606-8 20169472 PMC2852520

[35] De Vet HC, Terwee CB, Mokkink LB, Knol DL. Measurement in medicine: a practical guide: Cambridge University Press; 2011.

[36] Mokkink LB, Terwee CB, Knol DL, Stratford PW, Alonso J, Patrick DL, et al. The COSMIN checklist for evaluating the methodological quality of studies on measurement properties: a clarification of its content. BMC medical research methodology. 2010;10:22. Epub 2010/03/20. 10.1186/1471-2288-10-22 20298572 PMC2848183

[37] Cohen J. Statistical power analysis for the behavioral sciences. 2nd ed. Hillsdale, NJ: Erlbaum; 1988.

[38] Dunn KM, Croft PR. Classification of low back pain in primary care: using "bothersomeness" to identify the most severe cases. Spine. 2005;30(16):1887–92. Epub 2005/08/17. 10.1097/01.brs.0000173900.46863.02 . 16103861

[39] Terwee CB, Bot SD, de Boer MR, van der Windt DA, Knol DL, Dekker J, et al. Quality criteria were proposed for measurement properties of health status questionnaires. Journal of clinical epidemiology. 2007;60(1):34–42. Epub 2006/12/13. 10.1016/j.jclinepi.2006.03.012 . 17161752

[40] Bier JD, Ostelo R, Koes BW, Verhagen AP. Validity and reproducibility of the modified STarT Back Tool (Dutch version) for patients with neck pain in primary care. Musculoskeletal science & practice. 2017;31:22–9. Epub 2017/06/24. 10.1016/j.msksp.2017.06.006 . 28644962

[41] Taft C, Karlsson J, Sullivan M. Do SF-36 summary component scores accurately summarize subscale scores? Quality of life research: an international journal of quality of life aspects of treatment, care and rehabilitation. 2001;10(5):395–404. Epub 2002/01/05. 10.1023/a:1012552211996 . 11763202

[42] Hill JC, Afolabi EK, Lewis M, Dunn KM, Roddy E, van der Windt DA, et al. Does a modified STarT Back Tool predict outcome with a broader group of musculoskeletal patients than back pain? A secondary analysis of cohort data. BMJ open. 2016;6(10):e012445. Epub 2016/10/16. 10.1136/bmjopen-2016-012445 27742627 PMC5073547

[43] Terwee CB, Mokkink LB, Knol DL, Ostelo RW, Bouter LM, de Vet HC. Rating the methodological quality in systematic reviews of studies on measurement properties: a scoring system for the COSMIN checklist. Quality of life research: an international journal of quality of life aspects of treatment, care and rehabilitation. 2012;21(4):651–7. Epub 2011/07/07. 10.1007/s11136-011-9960-1 21732199 PMC3323819

[44] Landis JR, Koch GG. The measurement of observer agreement for categorical data. Biometrics. 1977;33(1):159–74. Epub 1977/03/01. . 843571

[45] Sim J, Wright CC. The kappa statistic in reliability studies: use, interpretation, and sample size requirements. Physical therapy. 2005;85(3):257–68. Epub 2005/03/01. . 15733050

[46] Newell D, Field J, Pollard D. Using the STarT Back Tool: Does timing of stratification matter? Manual therapy. 2015;20(4):533–9. Epub 2014/09/02. 10.1016/j.math.2014.08.001 . 25175750

[47] Hay EM, Dunn KM, Hill JC, Lewis M, Mason EE, Konstantinou K, et al. A randomised clinical trial of subgrouping and targeted treatment for low back pain compared with best current care. The STarT Back Trial Study Protocol. BMC musculoskeletal disorders. 2008;9:58. Epub 2008/04/24. 10.1186/1471-2474-9-58 18430242 PMC2377248

[48] Beneciuk JM, Fritz JM, George SZ. The STarT Back Screening Tool for prediction of 6-month clinical outcomes: relevance of change patterns in outpatient physical therapy settings. The Journal of orthopaedic and sports physical therapy. 2014;44(9):656–64. Epub 2014/08/08. 10.2519/jospt.2014.5178 . 25098194

[49] Morso L, Kent P, Albert HB, Hill JC, Kongsted A, Manniche C. The predictive and external validity of the STarT Back Tool in Danish primary care. European spine journal: official publication of the European Spine Society, the European Spinal Deformity Society, and the European Section of the Cervical Spine Research Society. 2013;22(8):1859–67. Epub 2013/02/12. 10.1007/s00586-013-2690-z 23397189 PMC3731474

[50] Tack C. Artificial intelligence and machine learning | applications in musculoskeletal physiotherapy. Musculoskeletal science & practice. 2019;39:164–9. Epub 2018/12/07. 10.1016/j.msksp.2018.11.012 . 30502096

[51] Alyass A, Turcotte M, Meyre D. From big data analysis to personalized medicine for all: challenges and opportunities. BMC medical genomics. 2015;8:33. Epub 2015/06/27. 10.1186/s12920-015-0108-y 26112054 PMC4482045

[52] Prosperi M, Min JS, Bian J, Modave F. Big data hurdles in precision medicine and precision public health. BMC medical informatics and decision making. 2018;18(1):139. Epub 2018/12/31. 10.1186/s12911-018-0719-2 30594159 PMC6311005

[53] Ngiam KY, Khor IW. Big data and machine learning algorithms for health-care delivery. The Lancet Oncology. 2019;20(5):e262–e73. Epub 2019/05/03. 10.1016/S1470-2045(19)30149-4 . 31044724

[54] Bujang MA, Adnan TH. Requirements for Minimum Sample Size for Sensitivity and Specificity Analysis. Journal of clinical and diagnostic research: JCDR. 2016;10(10):Ye01–ye6. Epub 2016/11/29. 10.7860/JCDR/2016/18129.8744 27891446 PMC5121784

[55] Schober P, Boer C, Schwarte LA. Correlation Coefficients: Appropriate Use and Interpretation. Anesthesia and analgesia. 2018;126(5):1763–8. Epub 2018/02/27. 10.1213/ANE.0000000000002864 . 29481436

[56] Portney LG, Watkins MP. Foundations of Clinical Research: applications to practice. 3th ed. Upper Sadle River, NJ: Prentice Hall Health; 2014.

[57] Protheroe J, Saunders B, Bartlam B, Dunn KM, Cooper V, Campbell P, et al. Matching treatment options for risk sub-groups in musculoskeletal pain: a consensus groups study. BMC musculoskeletal disorders. 2019;20(1):271. Epub 2019/06/04. 10.1186/s12891-019-2587-z 31153364 PMC6545223

