# Correction: Validity and reliability of the Dutch STarT MSK tool in patients with musculoskeletal pain in primary care physiotherapy

**DOI:** 10.1371/journal.pone.0252912

**Published:** 2021-06-03

**Authors:** The PLOS ONE Staff

## Notice of republication

An incorrect version of S1 Appendix and S1 File was published in error. References to these Supporting Information have been updated throughout the article. The updated S1 and S2 Appendix were the previous S2 and S3 Appendix. This article was republished on May 28, 2021 to correct these errors. Please download this article again to view the correct version.
