# Supplementary material for: Validity and reliability of the Dutch STarT MSK tool in patients with musculoskeletal pain in primary care physiotherapy
Source: PLoS One. 2021 Mar 18;16(3):e0248616. doi: 10.1371/journal.pone.0248616 (PMC7971537; doi:10.1371/journal.pone.0248616)
Supplement: S1 Appendix — (PDF) [file pone.0248616.s001.pdf]

S1 Appendix. Forward-backward translation method Dutch STarT MSK tool.

Two native Dutch speakers independently performed a forward translation. Based on a consensus meeting a single preliminary Dutch version was formed. This version was translated back into English independently by two native English speakers with no medical background. An expert committee consisting of two forward translators, one backward translator, two clinical health scientists and one physiotherapist then reviewed the original STarT MSK tool and each translated version, which resulted in a pre-final version of the Dutch STarT MSK tool. Finally, this pre-final version was tested in a pilot consisting of 20 Dutch-speaking patients who consulted a physiotherapist for musculoskeletal pain. After completing the questionnaire, patients were briefly interviewed about the interpretation and comprehension of each item and the chosen response. As no problems were reported, the pre-final version of the Dutch STarT MSK tool was considered final.
