# Supplementary material for: Validity and reliability of the Dutch STarT MSK tool in patients with musculoskeletal pain in primary care physiotherapy
Source: PLoS One. 2021 Mar 18;16(3):e0248616. doi: 10.1371/journal.pone.0248616 (PMC7971537; doi:10.1371/journal.pone.0248616)
Supplement: S2 Appendix — (PDF) [file pone.0248616.s002.pdf]

S2 Appendix. The Dutch version of the STarT MSK tool (including scoring method).

Naam: ____ Datum: _______________

Denk bij het beantwoorden van de vragen 1-9 alleen aan de laatste 2 weken:

Pijnintensiteit

1. Gemiddeld genomen, hoe hevig was uw pijn (waarbij 0 betekent "geen pijn" en 10 "ergste pijn denkbaar")?

**0**	**1**	**2**	**3**	**4**	**5**	**6**	**7**	**8**	**9**	**10**
□	□	□	□	□	□	□	□	□	□	□
0	0	0	0	0	1	1	2	2	3	3

	Kruis alstublieft één vakje aan bij elke onderstaande vraag	**Ja**<br>1	**Nee**<br>0
2.	Voelt u zich vaak onzeker over hoe u met uw pijn moet omgaan?	□	□
3.	Bent u in de laatste 2 weken veel gehinderd door uw pijn?	□	□
4.	Bent u door uw pijn alleen in staat geweest korte afstanden te lopen?	□	□
5.	Heeft u hinderlijke gewrichts- of spierpijn gehad in meer dan één lichaamsdeel?	□	□
6.	Denkt u dat uw klacht lang zal aanhouden?	□	□
7.	Heeft u andere belangrijke gezondheidsproblemen?	□	□
8.	Heeft u zich in de laatste 2 weken somber of depressief gevoeld door uw pijn?	□	□
9.	Heeft u het gevoel dat het voor iemand met uw klacht onveilig is om lichamelijk actief te zijn?	□	□
10.	Heeft u uw huidige pijnklacht sinds 6 maanden of langer?	□	□

**Totaalscore:**	0-4 = Laag risico<br>□	5-8 = Gemiddeld risico<br>□	9-12 = Hoog risico<br>□
