# Supplementary material for: Validity and reliability of the Dutch STarT MSK tool in patients with musculoskeletal pain in primary care physiotherapy
Source: PLoS One. 2021 Mar 18;16(3):e0248616. doi: 10.1371/journal.pone.0248616 (PMC7971537; doi:10.1371/journal.pone.0248616)
Supplement: S1 Table — (PDF) [file pone.0248616.s003.pdf]

S1 Table. Sensitivity analysis predictive validity using different subgroup cut-points.*

	Proportion (95% CI)	Relative risk (95% CI)[¶]
Low risk (0-3)[‡]	15.0 (8.1-26.1)	NA
Medium risk (4-7)[‡]	22.4 (14.1-33.7)	1.49 (0.71-3.16)
High risk (8-12)[‡]	77.8 (45.3-93.7)	5.19 (2.59-10.40)
Low risk (0-4)[₸]	13.7 (7.6-23.4)	NA
Medium risk (5-7)[₸]	25.9 (16.1-38.9)	1.89 (0.91-3.93)
High risk (8-12)[₸]	77.8 (45.3-93.7)	5.68 (2.90-11.13)

* Proportions and relative risks of persistent disability at 3 months. Persisting disability is defined as a Physical Functioning scale score equal to or below the baseline median at 3 months.

‡ Low risk (n = 60); Medium risk (n = 67); High risk (n = 9).

₸ Low risk (n = 73); Medium risk (n = 54); High risk (n = 9)

¶ Relative risks for medium risk and high risk, each compared with low risk.

CI = confidence interval; NA = not applicable.
